# Early growth response-1, a dynamic conduit in cardiovascular disease

**DOI:** 10.3389/fcvm.2024.1487668

**Published:** 2024-11-15

**Authors:** Levon M. Khachigian

**Affiliations:** Vascular Biology and Translational Research, Department of Pathology, Faculty of Medicine and Health, School of Biomedical Sciences, University of New South Wales, Sydney, NSW, Australia

**Keywords:** cardiovascular disease, early growth response-1, EGR1, acute coronary syndrome, vascular biology

## Abstract

The transcription factor, early growth response-1 (Egr-1) is the product of a prototypic immediate-early gene that plays an integral role in the pathogenesis of multiple cardiovascular diseases. Egr-1 has been linked with atherogenesis, myocardial ischemia-reperfusion injury, cardiac fibrosis and heart failure. Egr-1 expression is triggered by a host of factors including cytokines, hormones, growth factors, hyperglycaemia, biomechanical forces and oxygen deprivation. Egr-1 is a molecular conduit that links changes in the cellular environment with the inducible expression of genes whose products play a causative role in this inflammatory disease. It is rapidly synthesised, undergoes post-translational modification, interacts with a range of cofactors and drives gene expression. Studies in Egr-1 deficient mice, animal models using DNAzymes, RNA interference, oligodeoxynucleotide decoys, antisense oligonucleotides, and new insights provided by technologies such as single cell RNA sequencing, have shaped our understanding of the importance of Egr-1 in the initiation and progression of cardiovascular disease. This article describes Egr-1's role in various cardiovascular settings and discusses potential mechanisms of action. Given the range of conditions linked to Egr-1, this zinc finger protein may serve as a therapeutic target for intervention.

## Introduction

Cardiovascular disease remains the leading cause of death worldwide (1). In 2020, approximately one-third of adults in the United States received care for a cardiovascular risk factor or condition (2). This public health burden is set to increase. For example, health care costs to manage cardiovascular risk factors are expected to rise dramatically, from $400 billion in 2020 to $1,344 billion in 2050 (2) and productivity losses are expected to rise by 54% from $234 billion to $361 billion.

Transcription factors regulate the expression of genes underpinning the pathogenesis of cardiovascular disease by binding to DNA regulatory elements, interacting with other regulatory proteins and undergoing modification themselves. Early growth response (Egr-1/EGR1) is a zinc finger transcription factor and product of an immediate-early gene which rapidly responds to changes in the cellular environment. Egr-1 has been linked with multiple cardiovascular disorders including reperfusion injury, myocardial fibrosis, no reflow and heart failure.

**Figure 1:**
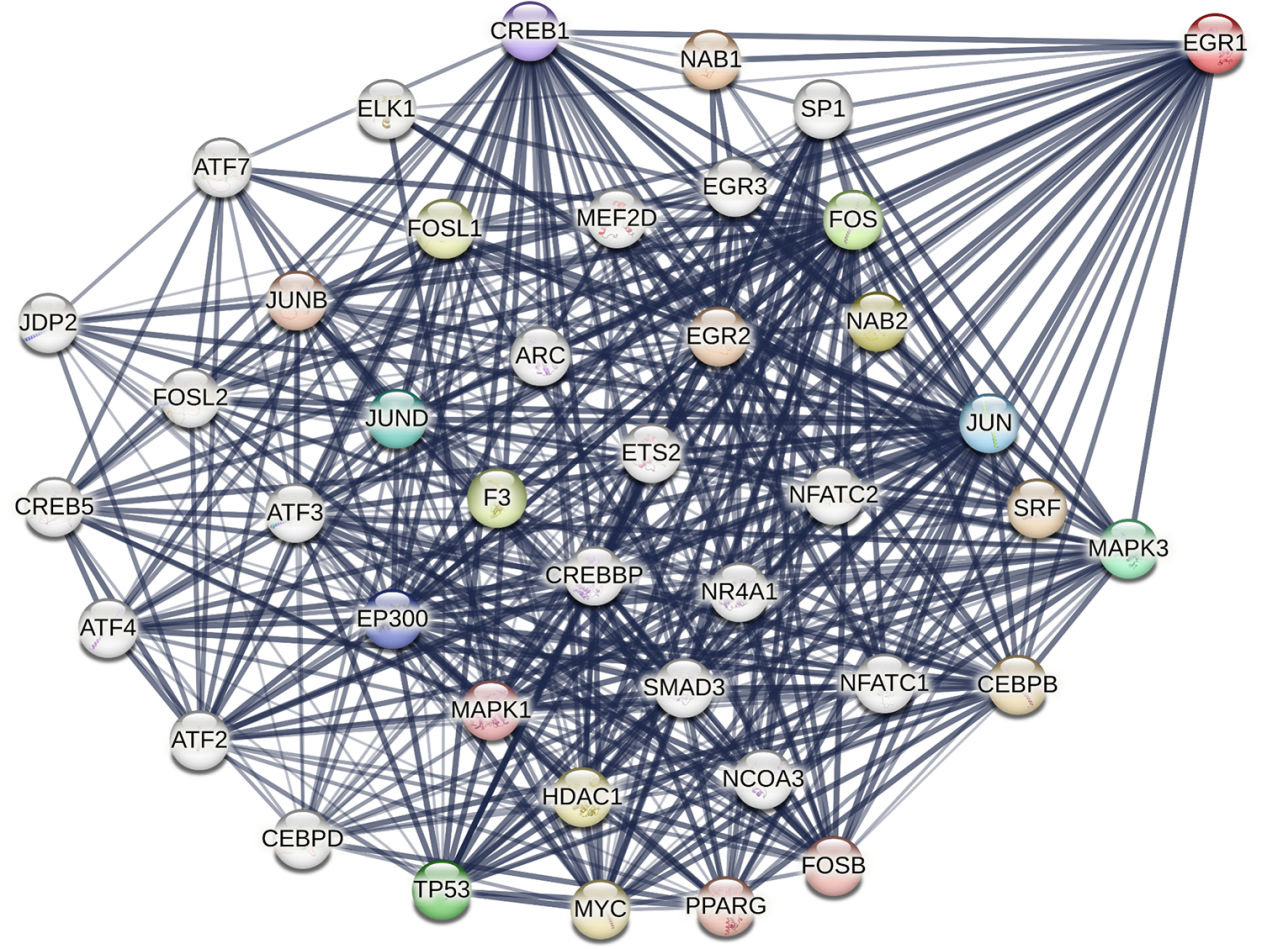
STRING representation of structural and/or functional interactions between human EGR1 with other proteins. Image was created in STRING (Protein-Protein Interaction Networks Functional Enrichment Analysis) using the full STRING network setting with edges indicating functional and physical protein associations in confidence mode, sourcing text, experiments and databases, and relative line thickness indicating strength of data support. Interactions limited to 40.

Egr-1 was discovered 3 decades ago and comprises N-terminal and C-terminal activation domains, several C_2_H_2_ type DNA-binding zinc fingers, nuclear localization signals and an inhibitory domain (3–8). Egr-1 functionally interacts with a broad range of partners, including protein kinases (*e.g.,* MAPK3/ERK1), transcriptional activators [*e.g.,* C/EBPβ (9), JUN (10)] and transcriptional repressors [*e.g.,* NGFI-A binding protein 1 (NAB1) (11) and NAB2 (12)] (Figure 1). These cofactors have been found to play a causal role in the development of cardiovascular dysfunction. For example, ERK1 phosphorylates EGR1 (at Ser26), an amino acid in EGR1 critical for vascular endothelial cell proliferation and migration (13, 14). EGR1's interaction with C/EBPβ controls transcription of the human low-density lipoprotein (LDL) receptor gene (9), which is linked with hypercholesterolemia and cardiovascular disease (15). Cardiac-specific NAB1 overexpression regulates cardiomyocyte growth through interaction with Egr-1 and inhibits cardiac hypertrophy in response to pressure overload (16). Recent work indicates that EGR1 can also interact with chromatin remodeling proteins, including subunits of the nucleosome remodeling and deacetylation (NuRD) complex to repress inflammatory enhancers in macrophages and plays a gatekeeper role in monocytic commitment (17). Trizzino et al. correlated EGR1 binding with enhancer activation and repression during macrophage differentiation. On one hand, EGR1 transactivates through direct interactions with its DNA motif, while on the other, appears to act as a corepressor at myeloid enhancers enriched with AP1, PU.1, C/EBP*α* and other macrophage factors. EGR1 was found to be required for the repression of approximately 1,600 enhancers in the course of macrophage differentiation (17). Recent work by Ding et al*.* in cancer cells shows that EGR1 can directly bind HDAC9 (18), a histone deacetylase that promotes vascular inflammation and atherosclerosis (19).

This article briefly reviews emerging evidence further implicating Egr-1 in the pathogenesis of cardiovascular disease.

## Egr-1, atherosclerosis and acute coronary syndrome

Building on pioneering studies from a range of laboratories that established pro-inflammatory and pro-atherogenic roles for Egr-1 (20, 21), Manta and colleagues (22) studied links between Egr-1 and stabilin proteins in atherosclerosis using *Apoe^−/−^* mice interbred with *stab1^−/−^* and *stab2^−/−^* mice. Stab1 and stab2 are scavenger receptors expressed in the liver by sinusoidal endothelial cells implicated in atherogenesis. Low-density lipoprotein and ox-LDL uptake into caudal veins are reduced *stab1^−/−^stab2^−/−^* zebrafish (23). Manta et al. (22) found that atherosclerotic plaque burden was substantially lower in *Apoe^−/−^stab1^−/−^* and *Apoe^−/−^stab2^−/−^* animals, and that monoclonal antibodies targeting stab1 or stab2 reduced diet-associated atherosclerosis in *Apoe^−/−^*mice and *Ldlr^−/−^*mice. Single-cell RNA seq of circulating myeloid cells from *Apoe^−/−^*, *Apoe^−/−^stab1^−/−^* and *Apoe^−/−^stab2^−/−^* mice revealed transcriptional changes in patrolling (Ccr2^–^/Cx3cr1^+^/Ly6C^lo^) and inflammatory (Ccr2^+^/Cx3cr1^+^/Ly6C^hi^) monocytes, including reduced expression of Egr-1. Egr-1 was the only gene downregulated in both monocyte subtypes (22). scRNA-seq by Cochain et al*.* showed that *EGR1* (along with *PHLDA1, ATF3, NLRP3* and *CCL2*) is expressed in macrophages of both human and mice atherosclerotic lesions (24). Peripheral blood cytospin staining with anti-Cd11b and anti-Egr-1 antibodies confirmed downregulation of Egr-1 in Cd11b^+^ cells of *Apoe^−/−^stab1^−/−^* and *Apoe^−/−^stab2^−/−^* mice compared with those from *Apoe^−/−^* mice. Egr-1 attenuation may represent a common mechanism for lower proatherogenic activation of monocytes. Atheroprotection resulting from stab-deficiency is likely due to Egr-1 downregulation in monocytes by plasma proteins regulated by stabilin-reliant clearance.

Egr-1 is also thought to play a role in plaque thrombogenicity and instability, which underpin acute myocardial infarction. Egr-1 activates tissue factor expression in macrophages (25, 26). Tissue factor expression is inhibited by simvastatin in advanced atherosclerotic lesions of *Apoe^−/−^*mice (27). Recent studies by Severino et al*.* (28) investigated the effects of atorvastatin on CD4 ^+^ T-cells in statin-naïve non-ST elevation acute coronary syndrome patients. Atorvastatin reduced levels of *EGR1* (−10.9 fold) (most profound inhibition) and *FOS* (−5.5 fold), and decreased the proportion of CD4^+^CD28^−^T-cells producing IFN-γ (from 44.1% to 15.0%). CD4^+^CD28^−^T-cells have been implicated in weakening of the fibrous cap and atherosclerotic plaque rupture (29). Interestingly, atorvastatin can reduce levels of tissue factor, an EGR1-dependent gene (30), in cholesterol-fed rabbits (31). Moreover, atorvastatin decreased levels of pro-inflammatory cytokines IL-6 and IL-18, chemokine (C-C motif) receptor-2 (CCR2) and Toll-like receptor-4 (TLR4). The authors surmised atorvastatin's inhibitory effects on EGR1 may account for why the statin reduces inflammation in acute coronary syndromes. Future studies should determine whether other statins have similar effects on EGR1 and tissue factor in this setting. Additionally, Wang et al. (32) reported the involvement of Egr-1 in coronary microembolization (CME), a thrombotic and microinfarction complication that can arise in acute coronary syndrome patients during percutaneous coronary intervention (PCI) and result in no reflow. In a rat model of CME, in which myocardial tissue underwent edema and inflammatory cell infiltration around injected microspheres, the authors found that Egr-1 shRNA reduced myocardial injury (serum cardiac troponin I levels) and microinfarct area. Moreover, qPCR after CME-induced myocardial injury revealed activation of the Egr-1/Bim/Beclin-1 pathway impacting autophagy and apoptosis. Egr-1 silencing increased LC3-II and Beclin-1 levels and ameliorated levels of cleaved caspase-3, p62 and Bim. Future studies using alternatives to plastic microspheres to cause a coronary microembolus would better reflect the clinical situation and build on these important proof-of-principle investigations.

Apoptosis underpins myocyte loss after acute myocardial infarction, which can lead to LV remodeling and heart failure (33). Recent studies by Zhou et al*.* revealed that EGR1 stimulates the expression of a range of apoptosis-related proteins (ATF2, ELK1, HAND2 and CTCF) to promote cardiomyocyte cell death. Mutation of Ser501 (to Ala) reduced EGR1 phosphorylation and impaired the pro-apoptotic effect of EGR1 in cardiomyocytes in a JNK-dependent manner (34).

Biomechanical forces impact atherosclerotic lesion formation and progression. Oscillatory shear stress (OSS) is an atheroprone biomechanical force that can induce the expression of endothelial cell surface adhesion molecules, such as VCAM-1 and ICAM-1, which facilitate leukocyte adhesion and invasion into the arterial wall (35). Egr-1 is activated in endothelial cells by fluid shear stress (36), a response inhibited by PD98059 indicating control via the MEK/ERK pathway (37). Bondareva et al*.* recently found that EGR1 was induced by OSS and its binding motif is among the most prominent enriched motifs (38). This followed work by Ajami et al*.* using pathway analysis to identify an SRF-EGR1-HIF1A regulator*y* axis for OSS up-regulation and that a range of mediators including cytokines, focal adhesions and eNOS activation are targets of EGR1 (39). Egr-1 modulation by biomechanical forces has recently been exploited in a bioreactor application using a non-endothelial system. Kwon et al*.* developed CHO-DG44 cells that express GFP in small-scale bioreactors secondary to EGR1 promoter activation by shear stress although the definition of an exact shear stress value was not achieved in their sensor design (40).

## Egr-1, cardiomyopathy and fibrosis

The adult heart lacks the capacity to regenerate and heart failure can ensue after cardiac ischemia and fibrosis (41). Li et al*.* (42) sought to identify potential targets for vascular regeneration by investigating the transcriptomic dynamics of coronary vascular endothelial cells following ischemic injury in the heart through comprehensive meta-analysis of publicly available single-cell RNA-sequencing data. *Egr-1/EGR1* was among the most significantly upregulated genes in human and murine vascular endothelial cells after ischemia (along with *Jun*, *Zfp36*, *Fosb*, and *Hsp90aa1)*, findings supported by substantially greater EGR1^+^CD31^+^ double staining in coronary tissue from patients with ischemic cardiomyopathy compared with normal hearts (42). Cardiac ischemia and fibrosis are primary causes of end-stage heart failure (43). Egr-1 is induced by profibrotic stimuli such as TGF-β and can promote collagen synthesis, extracellular matrix production and fibrosis (44, 45).

Koenig et al*.* (46) used scRNA-seq and single-nucleus (sn)RNA-seq on left ventricular cardiac tissue from 17 individuals with dilated (non-ischemic) cardiomyopathy (DCM) and 28 non-diseased donors. *EGR1* was one of the most significantly upregulated genes in vascular endothelial cells (alongside *DUSP5/6, PDE4B/D, FGFR1, SMAD3/6, VEGF-A/C, APLNR*) and macrophages, monocytes and dendritic cells (along with *CCL3, NLRP3, NFKB2*) in DCM material compared to non-diseased tissue. While this study sheds light on the transcriptional landscape of cells in healthy and diseased human heart, future experiments exploring signaling mechanisms or the potential role of chromatin modification and accessibility involving EGR1 in this context would provide added mechanistic insights on molecular control in the pathogenesis of heart failure.

Investigations by Shen et al*.* showed that inhibition of *Egr1* expression in mice relieved the severity of myocardial fibrosis, whereas delivery of miR-150-5p antagomir exacerbated myocardial fibrosis, reversing the effect of sh-*Egr1* (47). A recent study by Zhang et al. (48) showed that Egr-1 regulates regenerative senescence during neonatal heart regeneration as well as cardiac repair in adult mice. In neonatal hearts, Egr-1 mediates angiogenesis and cardiomyocyte replication, whereas in adult hearts, senescence and repair induced by the heparan sulfate proteoglycan, agrin, involves integrin–FAK–ERK–Akt1 signaling and Egr-1 activation in cardiac fibroblasts. The authors found increased fibrotic scar formation and impaired cardiac function in Egr-1 deficient mice treated with agrin compared with wild-type mice, demonstrating that Egr1 is needed for agrin-induced cardiac repair. Future work should delineate whether cellular localisation impacts Egr-1 function and use conditional Egr-1 deficient mice to better understand Egr-1 mediated senescence in cardiac fibroblasts during repair.

## Egr-1 and vascular dysfunction

Tian et al*.* (49) reported EGR1 regulation of sushi, von Willebrand factor type A, EGF, and pentraxin domain-containing protein 1 (SVEP1), a large extracellular matrix protein associated with coronary artery disease (50). In *Apoe^−/−^* or *Svep1^+/−^Apoe^−/−^* mice fed a high fat diet for 8 weeks, *Svep1^+/−^Apoe^−/−^* mice had reduced atherosclerotic plaque burden. Moreover, *Svep1* deficiency specifically in SMC reduced atherosclerotic and plaque size and complexity (51). Levels of SVEP1, which mediates SMC migration and proliferation in response to ox-LDL, are elevated in human atherosclerotic plaques compared to normal tissues. EGR1 binds to and transactivates the SVEP1 promoter and drives SVEP1 expression in SMCs treated with ox-LDL (49). Separate studies by Xie et al. (52) revealed that the splicing factor, serine/arginine-rich splicing factor 1 (SRSF1) promotes SMC proliferation and injury-induced neointima formation and involves induction of truncated form of p53, Δ133p53, which activates Krüppel-like factor 5 (KLF5), a process requiring the physical interaction of Δ133p53 and EGR1. These findings suggest that Δ133p53-EGR1 complex formation is needed for SRSF1-inducible KLF5 signalling and SRSF1-dependent proliferation (52). That Egr-1 promotes SMC hyperplasia has been demonstrated using a range of approaches (53–63). For example, intimal thickening is reduced when blood vessels from *Egr-1*-deficient mice are injured compared with wild type animals (64) and lumens of vein grafts from *Egr-1*^−/−^ mice are wider than those of wild types (65). Tian et al*.* and Xie et al.'s data suggest that the growth-regulatory effects of EGR1 on SMCs are reliant on SVEP1, SRSF1 and/or other genes. For example, Mylonas et al*.* (66) reported in a rabbit vein graft model that Egr-1 oligodeoxynucleotide decoys that reduce the expression of pro-inflammatory transcription factors (*NF-**κ**B, KLF4, HIF1α*), stem cell genes (*NANOG* and *HOXA5*), toll-like receptors (*TLR2, TLR3, TLR4, TLR8*), chemokines (*CCL4, CCL20, CCR2*), interleukins (*IL1β, IL2, IL4, IL8, IL10, IL18*), TNF*α* and interferons (*IFNβ, IFN*γ). These decoys reduce Egr-1 expression, ki-67^+^ proliferation and intimal hyperplasia (67). Egr-1 is thus an attractive target to control intimal thickening given the breadth and nature of genes it regulates.

## Egr-1 in hypertension and preeclampsia

The direct relationship between hypertension and the risk of cardiovascular disease is well established (68, 69). Systolic blood pressure is a leading modifiable risk factor for premature cardiovascular death (70). EGR1 levels are elevated in advanced vascular lesions from individuals with late-stage pulmonary arterial hypertension (71). Laggner et al*.* recently implicated EGR1 as a driver of right ventricular remodeling in idiopathic pulmonary arterial hypertension and chronic thromboembolic pulmonary hypertension patients (72). Preeclampsia is a common complication in pregnancy and defined by onset of hypertension, proteinurea and organ failure after 20 weeks’ gestation. The pathogenesis of preeclampsia is incompletely understood but is associated with vasospasm and impaired placental angiogenesis. Zhao and colleagues (73) studied serum fibroblast growth factor 23 (FGF23) in healthy women or pregnant women with preeclampsia and examined its role mediating placental angiogenesis through ERK1/2-EGR1 signaling. They found that third trimester FGF23 levels were lower in women with preeclampsia. FGF23 stimulated endothelial cell migration, invasion and tubule formation *in vitro.* It also promoted placental VEGF-A expression through the ERK1/2-EGR-1 pathway since SCH772984 blocked both EGR1 and VEGF-A in these cells. Moreover, levels of p-ERK, EGR1 and VEGF-A in placental tissue were lower in the preeclampsia group than in controls. Whether Egr-1 directly regulates VEGF-A transcription in endothelial cells appears to be context-dependent since VEGF-A levels do not appear to change at least in retinas of mice deficient in Egr-1 (74) or when Egr-1 is knocked down (75) or overexpressed (76). While Zhao et al*.*'s studies suggest a potential mechanism in the development of preeclampsia, future studies should determine if FGF23 levels correlate with preeclampsia severity or if the FGF23-ERK1/2-EGR-1 pathway is causal for preeclampsia in animal models (73).

## Egr-1 and complications of diabetes

Egr-1 plays a causal role in the development of type 2 diabetes. Shen and colleagues (77) found that glucagon, which increases blood glucose levels by regulating gluconeogenesis, stimulates Egr-1 which binds the promoter of the gluconeogenic transcription factor C/EBP*α* and activates the expression of hepatic gluconeogenic genes. Xu et al*.* reported that Egr-1 drives the development of renal tubulointerstitial fibrosis in the context of diabetic kidney disease. Egr-1 activates the expression of renal fibrosis markers (such as protease-activated receptor 1, TGF-β1, fibronectin and collagen I) via the TGF-β1/Smad pathway (78). Recent work comparing differential gene expression across multiple autoimmune disorder datasets, including type 1 diabetes, revealed that EGR1 was the only gene commonly expressed across four datasets (type 1 diabetes, rheumatoid arthritis, systemic lupus erythematosus, Crohn's disease) (79). Beyond type 2 diabetes, which mainly affects older individuals, EGR1 has been used as a marker gene for childhood-onset type 2 diabetes (80).

Diabetic retinopathy is a major cause of vision loss and disability. Ao et al*.* (81) found that Egr1 is highly expressed in retinas of hyperglycaemic streptozotocin (STZ)-treated diabetic rats. Intravitreal delivery of *Egr-1* shRNA to STZ-induced diabetic rats reduced Egr-1 and p53 levels and inhibited apoptosis in the retina. Karthikkeyan et al*.* showed that EGR1 is also expressed human diabetic retina compared with non-diabetic retina (82). Moreover, El-Asrar et al*.* showed EGR1 expression in epiretinal membranes from individuals with proliferative diabetic retinopathy (mainly by vascular endothelial cells and stromal cells) and proliferative vitreoretinopathy (mainly myofibroblasts). EGR1 expression was coincident with HMGB1, RAGE, and OPN expression, suggesting its involvement in vitreoretinal angiogenic, inflammatory and fibrotic processes (83).

## Future research directions

The studies above have expanded our appreciation of the regulatory role that Egr-1 plays in vascular disease. Several limitations have already been identified that need to be addressed to advance the field. Additionally, many articles have described Egr-1 function without clarifying direct molecular mechanisms in a specific cardiovascular disease. For example, while Egr-1 is a well-established prototypic transcription factor, does it functionally collaborate with other such factors and if so, which ones, how and in which cell types? Can single cell- or single nuclear-RNA-sequencing extend our appreciation of Egr-1 control in complex cardiovascular tissue? Does Egr-1 undergo post-translational modification(s), dynamic or otherwise, in the context of a specific cardiovascular disease? How can ERK-dependent Egr-1 promoter activation be studied separately from ERK-dependent Egr-1 phosphorylation in the context of cardiovascular disease when Egr-1 is expressed so rapidly? Moreover, it is vital that new EGR1-targeting agents be developed or existing approaches be refined, whether this be with natural, novel or repurposed pharmaceuticals or those that exploit nucleic acid strategies. In this regard, recent studies indicate that the naturally occurring agent astragaloside IV inhibits cardiac hypertrophy through ERK and PKCβII/Egr-1 activation in rodents born from mothers with intrauterine hypoxia (84). This can also include DNAzymes targeting EGR1, which have been used by ourselves and others in a range of local delivery models to reduce post-angioplasty restenosis (53, 54, 85), in-stent restenosis (54) and to lower infarct size following myocardial ischemia-reperfusion (86, 87). However, an important challenge has been overcoming low efficient tissue uptake after systemic delivery (88). Future studies could apply insights from other fields to models of cardiovascular disease. For example, Jiang et al*.* (89) encapsulated EGR1 DNAzymes (75) with poly(ethylenimine) and an amorphous Mn^2+^/Zn^2+^-coordinated inositol hexaphosphate (IP6) capsule modified with a cRGD targeting peptide shell then injected these intravenously into mice bearing tumors which accumulated the DNAzyme. cRGD peptides confer targeting ability via recognition of *α*_v_β_3_ integrin receptors, mimicking integrin-mediated endocytic cell entry. Upon endocytosis, the Mn^2+^/Zn^2+^-IP6 shell is degraded in the cell's acidic lysosomal environment releasing Mn^2+^, Zn^2+^ and the DNAzyme. These biocapsulated DNAzymes suppressed EGR1 levels in tumors and induced tumor cell death. Delivered intravenously, biocapsule DNAzymes inhibited EGR1 levels in, and growth of, pulmonary metastatic tumors (89). While this approach demonstrates the clinical potential of biocapsules for targeted inhibition of breast cancer, the same approach may be useful in vascular settings, especially given the crucial role played by *α*_v_β_3_ integrin in atherogenesis. For example, *α*_v_β_3_ antibodies inhibit macrophage infiltration into atherosclerosis lesions and lesion formation in diabetic pigs fed a high fat diet (90). Moreover, *α*_v_β_3_ mediates plaque angiogenesis, inflammation and SMC accumulation (91, 92) and positron emission tomography radiotracers (18F-fluciclatide) have targeted these as potential markers of atherosclerosis (93).

## Concluding remarks

As an immediate-early gene activated by a range of environmental cues such as cytokines, hormones, growth factors and hypoxia, Egr-1 integrates changes outside the cell with programmatic inducible gene expression. The breadth of dependent genes and scope of proliferative, migratory, immune and inflammatory conditions that EGR1 controls suggest this factor represents a fertile therapeutic target for drug development for many types of cardiovascular disease. A key challenge, however, is devising effective, clinically viable, preferably systemically deliverable interventional strategies that can selectively target EGR1 and provide sustained inhibition in cardiovascular settings involving complex, interactive molecular networks and comorbidities.
